# Increased incidence of autoimmune markers in patients with combined pulmonary fibrosis and emphysema

**DOI:** 10.1186/1471-2466-13-31

**Published:** 2013-05-22

**Authors:** Argyris Tzouvelekis, George Zacharis, Anastasia Oikonomou, Dimitrios Mikroulis, George Margaritopoulos, Anastasios Koutsopoulos, Antonis Antoniadis, Andreas Koulelidis, Paschalis Steiropoulos, Panagiotis Boglou, Matina Bakali, Marios Froudarakis, Demosthenes Bouros

**Affiliations:** 1 Department of Pneumonology, University Hospital of Alexandroupolis, Democritus University of Thrace, Alexandroupolis 68100, Greece; 2 Department of Radiology, University Hospital of Alexandroupolis, Democritus University of Thrace, Alexandroupolis, Greece; 3 Department of Cardiothoracic Surgery, University Hospital of Alexandroupolis, Democritus University of Thrace, Alexandroupolis, Greece; 4 Department of Pneumonology, General Hospital of Kavala, Kavala, Greece; 5 Department of Pathology, University Hospital of Alexandroupolis, Democritus University of Thrace, Alexandroupolis, Greece; 6 Department of Pneumonology, General Hospital of Serres, Serres, Greece; 7 Department of Microbiology, University Hospital of Alexandroupolis, Democritus University of Thrace, Alexandroupolis, Greece

## Abstract

**Background:**

Combined pulmonary fibrosis and emphysema (CPFE) is an umbrella term encompassing upper lobe emphysema and lower lobe pulmonary fibrosis with pathogenesis elusive. The aim of our study was to investigate the incidence of autoimmune markers in patients with CPFE.

**Methods:**

In this multicenter study we retrospectively evaluated records from patients with CPFE (n=40) and IPF (n=60) without emphysema. Baseline demographic characteristics, high-resolution computed tomography (HRCT), spirometry, histopathological, treatment, serum immunologic and survival data were investigated. B cell presence was estimated with CD20 immunostaining in representative lung biopsy samples from CPFE patients and control subjects.

**Results:**

A statistically significant increased number of CPFE patients with elevated serum ANA with or without positive p-ANCA titers compared to patients with IPF without emphysema was observed. Patients with CPFE and positive autoimmune markers exhibited improved survival compared to patients with a negative autoimmune profile. A massive infiltration of clusters of CD20+ B cells forming lymphoid follicles within the fibrotic lung in CPFE patients with positive serum immunologic profile compared to patients with negative profile, was noted and positively correlated with improved survival.

**Conclusions:**

A significant proportion of patients with CPFE may present with underlying auto-immune disorders that may reside insidiously and be associated with favorable prognosis. Early identification of these patients using a panel of auto-antibodies may lead to more targeted and effective therapeutic applications.

## Background

The combination of pulmonary fibrosis and emphysema (CPFE) is a recently defined syndrome, encompassing a distinct radiologic, revealing both upper lobe emphysema and lower lobe fibrosis in high resolution computed tomography (HRCT) of the chest, as well as lung function profile, with apparently preserved lung volumes contrasting with disproportionally impaired gas exchange, as assessed by reduced diffusing lung capacity for carbon monoxide (DLco) [1-3]. It is associated with severe exercise hypoxemia and increased prevalence of pulmonary hypertension, two major determinants of dismal prognosis, with a 1-year survival of only 60% if present and a median survival of 6.1 years if absent [4]. The syndrome of CPFE has been recently individualized within the spectrum of smoking-induced chronic lung diseases. In addition CPFE has been recently described in the context of connective tissue diseases [5] implicating autoimmunity in the pathogenesis of both pulmonary fibrosis and emphysema.

In the past, despite seminal reports pointing to an association between immune deregulation and paradigms of chronic lung injury [6], the role of autoimmunity in the pathogenetic cascade of both idiopathic pulmonary fibrosis (IPF) and chronic obstructive pulmonary disease (COPD) has been severely overlooked mainly due to the presence of a causal-effect relationship between smoking and COPD and the disappointing results of the current immunosuppressive and immunomodulatory agents in patients with IPF [7-10]. Nevertheless, interest in the role of autoimmunity in the pathophysiology of both IPF and COPD was revived by recent studies reporting highly activated and proliferative CD4+ cells [11] and global numerical and functional impairment of T regulatory cells [12], as well as presence of circulating auto-antibodies against nuclear and cytoplasmic antigens in both IPF and COPD patients [13,14]. Moreover, a close linkage between pulmonary fibrosis and microscopic polyangiitis (MPA), a type of systemic necrotizing small vasculitis characterized by both pulmonary and renal involvement and associated with circulating antineutrophil cytoplasm antibodies (ANCAs) against myeloperoxidase (MPO), has been recently identified in both clinical [15] and experimental setting [16]. The latter implies that an ongoing autoimmune process through recognition of self-antigens may take place in a subgroup of patients initially presented with a diagnosis of IPF. In line with this notion, a considerable number of patients seminally set under the diagnostic umbrella of idiopathic interstitial pneumonia (IIP), either non-specific (NSIP) or IPF meet the case definition of undifferentiated connective tissue disorder and may evolve through disease course into a specific connective tissue disorder with compatible clinical and serum immunologic profile [17,18].

Based on the above evidence, a significant proportion of both IPF and COPD patients present with a flare of autoimmunity that may reside occultly under the diagnostic “cover” of interstitial lung fibrosis and/or emphysema. Since CPFE presents with pathogenesis still elusive and controversial and it is debatable whether it represents a distinct syndrome facilitated by a common pathogenetic cascade leading to both fibrosis and emphysema in susceptible individuals after cigarette smoke exposure or it is just a phenotype of IPF with coincidental emphysema, we sought to determine the autoimmunity profile, using a panel of clinical, serum and histopathological markers, in a large cohort of patients with CPFE and correlate our findings with distinct survival patterns. Additionally our findings were compared to those observed in a cohort of patients with IPF without emphysema used as control group. In view of the current disappointing survival data arising from large prospective placebo-controlled clinical trials [19,20], identification of a subgroup of patients with CPFE with an autoimmunity background that could benefit from immunomodulatory treatment is of vital importance.

## Methods

### Study design

This multicentre study enrolled consecutive patients meeting the criteria for CPFE, as described by Cottin et al. [1] and patients with IPF according to the new ATS/ERS/JRS/ALAT 2011 criteria diagnosed in three different hospitals, (University Hospital of Alexandroupolis, General Hospital of Kavala and General Hospital of Serres, Greece), during the period of time between September 2004 and August 2010. The latter group served as control. Disease diagnosis was based on a multidisciplinary approach (chest physician, radiologist and pathologist). Clinical and serological data for cases included in the study were collected and analyzed on a retrospective basis. Patients were followed-up by August 2012 where the last data entry (clinical, serological, survival) in our retrospective analysis occurred. According to Greek legislation informed consent is not needed for retrospective analyses of data corresponding to current practice. This study was approved by Local Ethics Committee and the institutional review board of the University Hospital of Alexandroupolis, Democritus University of Thrace (579-03-2012).

### Serum immunologic profile

All patients (n = 100) enrolled in the study underwent a complete clinical and serum rheumatologic review based on the following criteria: based on the following criteria: 1) American College of Rheumatology (ACR) 1987 revised criteria for the classification of rheumatoid arthritis (RA) [21]; 2) the preliminary ACR criteria for systemic sclerosis (SSc) [22]; 3) the criteria proposed by Kahn et al. for mixed CTD [23]; 4) the American–European Consensus Group criteria for Sjogren’s syndrome [24]; and 5) the criteria for polymyositis and dermatomyositis described by Troyanov et al. [25]. Serum immunologic included the following: 1) antinuclear antibodies (ANA) 2) anti-double strand (anti-ds) DNA antibodies 3) anti-extractable nuclear antigens (ENA) panel including anti-scl70, anti-Ro (SSA), anti-La (SSB), anti-Sm, anti-RNP, 4) rheumatoid factor and anti-CCPs, 5) myositis panel including anti-Jo1 antibodies 6) complement 3 and 4 (C3,4) levels 7) antineutrophil cytoplasm antibodies (ANCAs) against myeloperoxidase (MPO) and proteinase-3 (PR-3). Assessment of the above panel of immunologic markers was performed at the following serial time points: 1) At time of diagnosis as a part of routine work-up in order to exclude collagen vascular diseases as a cause of lung fibrosis, and 2) At regular follow-up every 6 months or earlier than 6 months if clinical signs compatible with autoimmune disease (arthralgia, muscle weakness, dysphagia, Raynaud’s phenomenon, photosensitivity) or disease exacerbation (dyspnea deterioration, hemoptysis, hematuria) emerged.

### HRCT evaluation

All patients were subjected to HRCT of the thorax to set the diagnosis of CPFE and IPF. One mm thick- HRCT sections were acquired supine at full inspiration, at 10 mm intervals with a helical CT scanner (General Electric Prospeed Series). HRCT studies at the time of disease diagnosis were evaluated independently by 2 radiologists who were blinded to immunology and histopathology profile but were aware that the patients had CPFE syndrome. Discrepancies were resolved by consensus. HRCT studies were evaluated at five predetermined levels using a modification of a semiquantitative HRCT scoring system previously described [26]. HRCT scans were evaluated for the presence and extent of emphysema and the total extent of interstitial lung disease including the presence of reticular pattern, ground glass pattern and honeycombing. The above mentioned HRCT findings were analyzed and evaluated according to the Fleischner glossary of terms [27]. HRCT images were scored at five predetermined levels: 1) origin of great vessels; 2) main carina; 3) pulmonary venous confluence; 4) halfway between the third and fifth section; 5) immediately above the right hemi-diaphragm.

a) Extent of emphysema

The total extent of emphysema was estimated to the nearest five percent in each of the five sections, with global extent of emphysema on HRCT computed as the mean of the scores. To establish the diagnosis of CPFE on HRCT a global extent of emphysema higher than 5% was set as threshold.

b) Total extent of interstitial lung disease

The total extent of interstitial lung disease was estimated to the nearest five percent in each of the five sections, with global extent of disease on HRCT computed as the mean of the scores.

### Histopathology evaluation

Based on ATS/ERS/JRS/ALAT 2011 criteria [28] for IPF diagnosis video-assisted thoracoscopic surgery (VATS) procedure was performed and biopsy samples from two different lobes were obtained in selective number of cases in order to establish a more rigid diagnosis of IPF and to exclude other patterns of IIPs. Definite usual interstitial pneumonia (UIP) pattern was based on the presence of the following 4 criteria: 1) evidence of marked fibrosis, architectural distortion and honeycombing in a predominantly subpleural distribution, 2) presence of patchy involvement of lung parenchyma by fibrosis, 3) presence of fibroblastic foci, 4) absence of features against a diagnosis of UIP including hyaline membranes, organizing pneumonia, granulomas, predominant airway centered changes and marked interstitial inflammatory cell infiltrates away from honeycombing. Probable UIP pattern was defined by the presence of criteria numbers 1 and 4 and the absence of criteria numbers either 2 or 3, but not both. On the other hand NSIP histopathological diagnosis was defined as the presence of marked interstitial chronic inflammation, and/or interstitial fibrosis lacking the temporal heterogeneity pattern and the patchy features of UIP and the absence of fibroblastic foci [29].

### Tissue microarrays

Two tissue microarray blocks comprising of a total of 58 lung tissue samples including 15 lung fibrosis biopsy samples of different histopathologic patterns derived from patients with CPFE, 28 lung samples from patients with IPF and 15 control tissues extracted from the normal part of the lung removed for benign lesions, was constructed as previously reported [30]. Briefly, tissue cylinders of 2.0 mm diameter were punched from selected areas of each “donor” block by utilizing a thin-wall stainless tube from a precision instrument (TMA-100, Chemicon, USA) and were transferred by a solid stainless stylet into defined array coordinates in a 45 * 20 mm new recipient paraffin block. Each tissue element in the array was 2.0 mm in diameter and spacing between two adjacent elements was 0.1 mm. After the tissue microarray construction 3 μm sections for immunohistochemical analysis were cut from the “donor” blocks and were transferred to glass slides using an adhesive-coated tap sectioning system.

### Immunohistochemistry and semi-quantitative image analysis

Immunohistochemistry (IHC) was performed by using specific monoclonal antibody for CD20 (mouse anti-human, DAKO), as previously reported [31]. The number of CD20 positive cells in 5 fields per case was counted by two independent pathologists - observers using the high-resolution DUET, BioView scanning system for IHC morphology and immunocytochemistry applications, at ×100 magnification. All cell counts were expressed as cells/mm^2^.

### Statistical analysis

Statistical analysis was carried out using SPSS 14.0 software and Origin pro 8. Results are expressed as mean ± SD, or median (range), unless otherwise indicated. Pearson’s chi-square was used to compare frequencies of ANA and ANCA positivity among two studied populations (CPFE and IPF). Independent *t*-test was used to compare serological, pulmonary functional parameters between patients with CPFE and IPF, as well as CD20 positive cells/mm^2^ between CPFE and control lung samples. Spearman’s correlation was performed to find relationship between CD20 positive cells/mm^2^ and median survival in patients with CPFE and IPF. A p-value of < 0.05 was considered as statistically significant.

## Results

### Baseline characteristics

Baseline characteristics of patients enrolled in the study are demonstrated in Table 1. In total, 40 consecutive patients with CPFE and 60 patients with IPF were retrospectively identified from the archive records of three different hospitals, during the period of time between September 2004 and August 2010. Three patients with CPFE diagnosed with MPA based on clinical evidence (hemoptysis, hematuria), serum immunologic profile and renal biopsy showing pauci-immune necrotizing glomerulonephritis, were treated with pulses of methyprednisolone (1gr for 3 consecutive days) and cyclophosphamide (1gr for one day) and then, as maintenance treatment, with oral cyclophosphamide (60 mgr/day) and high doses of oral prednisolone (60 mg/day) that were gradually tapered, for a total period of 7 months (n = 3), as previously described[31]. One patient died due to respiratory and renal failure. The remaining 4 cases with CPFE presenting with positive p-ANCA and asymptomatic microscopic hematuria, with normal renal function and mild gas exchange impairment, evidence suggesting but not establishing the diagnosis of MPA, were switched from pirfenidone treatment to a more anti-inflammatory therapeutic regimen comprising of low doses of prednisolone (20 mgr/day), azathioprine (2 mg/kr/day) and high doses of NAC (1800 mgr/day).

**Table 1:** Baseline characteristics of the study population

**Characteristics**	**CPFE baseline data**	**IPF baseline data**
Subjects	40	60
Male	38	49
Age (yrs)	56 (31–74)	66 (44 – 78)
Current smokers	2	0
Ex-smokers	38	60
Steroid treatment	18	22
Bronchodilators	15	0
NAC	37	43
Pirfenidone	30	23
Cyclophosphamide	3	0
Azathioprine	7	11
VATS	15	28
FEV1 %predicted	68,6 ± 19	67,8 ± 9,1
FVC %predicted	70,6 ± 21*	62,8 ± 11,4
FEV_1_/FVC	77,6 ±11,2	83,2 ±10,1
TLC %predicted	69,7 ± 17,9*	57,1 ± 9,1
DL_CO_ %predicted	34,8 ±15*	44,1 ±7,2
MMEF25/75%predicted	69,3 ±35*	79,8 ±19
6MWD (m)	330 ± 168*	402 ± 112
sPAP (by echocardiography) mmHg	37,3 ± 14,5*	32,1 ± 10,9

Data are presented as median (range), No (total) or mean ± SD, unless stated otherwise. *p < 0.05 Abbreviations: *6MWD*: 6-minure walking distance, *DL*_*CO*_: Diffusing Capacity for carbon monoxide, *FVC*: Forced vital capacity, *MMEF*: maximum midexepiratory flow, *sPAP*: systolic pulmonary artery pressure, *TLC*: Total Lung Capacity.

Regarding pulmonary functional profile at the time of disease diagnosis, CPFE patients enrolled in our study exhibited a mild restrictive pattern with relatively preserved lung volumes and disproportionally impaired gas exchange and exercise capacity as indicated by DL_CO_ and 6-minute walking distance (6MWD) values (Table 1). Compared to patients with IPF, patients with CPFE demonstrated significantly higher FVC%pred and TLC%pred and lower DL_CO_, MMEF_25/75_%pred and 6MWD values (Table 1). Finally, patients with CPFE presented with a statistically significant elevated levels of systolic pulmonary artery pressure (sPAP) levels (37.3 ± 14.5 mmHg) compared to IPF patients (32.1 ± 10.9 mmHg) (p < 0.05), as assessed by cardiac ultrasonography (Table 2).

**Table 2:** Clinical, functional, radiological and histopathological characteristics of CPFE patients according to their immunologic profile

**Variable**	**ANA + ANCA+**	**ANA + ANCA-**	**ANA-ANCA-**
Total Number	7	10	23
FEV_1_%pred	69,2 ± 17,1	67,8 ± 14,3	68,4 ± 18,7
FVC%pred	72,87 ± 26,6	68,96 ± 22,9	69,54 ± 21,6
DL_CO_%pred	35,95 ± 12,3	35,79 ± 10,7	33,9 ± 10,4
Histopathology pattern	UIP	UIP	UIP
CD20 + cells/mm^2^	51.6 ± 6.9*	49.6 ± 7.1*	6.2 ± 3.6
Survival (median-range) months	51 (12 – 96)*		38 (16 – 61)
Radiology pattern	UIP	UIP	UIP
sPAP	36,9 ± 13,1	38,2 ± 12,8	37,1 ± 11,5
Hemoptysis	7	0	0
Hematuria	7	0	0
Arthralgias	0	3	0
Raynaud’s phenomenon	0	4	0
Dysphagia	0	1	0

Abbreviations: *ANA*: anti-nuclear antibodies, *ANCA*: antineutrophil cytoplasm antibodies (ANCAs), *DL*_*CO*_: Diffusing capacity of lung for carbon monoxide, *FVC*: Forced Vital Capacity, *sPAP*: systolic pulmonary artery pressure, *UIP*: Usual Interstitial Pneumonia. *p < 0.05.

HRCT findings showed that the mean global extent of emphysema was 15.7% (range: 5%-56%) and the mean global extent of interstitial lung disease was 42.2% (range: 6%-80%).

### Serum immunologic profile

All patients enrolled in the study were subjected to a complete serum immunologic profile. Strikingly there was a statistically significant increased number of CPFE patients presenting with elevated (>1/160) serum titers of ANA (n = 17/40, 42.5%) compared to patients with IPF (n = 16/60, 26.6%), (p < 0.05) (Table 3). Among these patients 15/17 (88%) exhibited positive ANA profile at the time of CPFE diagnosis, therefore were naïve of treatment, whereas in the remaining 2/17 (12%) ANA serum titers became positive after a mean period of 12 months following disease diagnosis. With regards to IPF subjects, 9/16 (56.2%) reported positive ANA profile at the time of diagnosis whereas the remaining 7/16 (43.8%) became positive after a mean follow-up period of 18 months. Furthermore, one of the most intriguing findings of our study was the identification of a higher proportion of CPFE ANA + patients that also exhibited positive ANCAs (n = 7/40, 17.5%) against MPO compared to IPF patients without emphysema (n = 0) (p < 0.05). Presence of autoantibodies was estimated by both qualitative (immunofluorescence) and quantitative (ELISA, mean levels = 15,6 elisa units –EU, normal range <9.0 EU) methods (Table 2). In 6 out of 7 patients ANA profile was positive and p-ANCA profile was negative at the time of CPFE diagnosis and became positive after several months (mean period of delay = 20 months) during either disease exacerbation (respiratory and renal failure) (n = 2) or in the setting of routine follow-up (n = 4). In one patient both ANA and p-ANCA concentrations were found elevated at the time when pulmonary and renal involvement were diagnosed. In 3 out of 7 CPFE patients diagnosis of MPA was firmly established by renal biopsy showing pauci-immune necrotizing glomerulonephritis while microscopic urine analysis revealed red blood cell renal casts. The latter group of patients exhibited the most aggressive clinical course comprising of respiratory and renal failure derivative of diffuse alveolar hemorrhage and necrotizing renal vasculitis. The remaining 4 CPFE patients with positive ANA and p-ANCA profile were characterized by an asymptomatic microscopic hematuria, with normal renal function and mild gas exchange impairment (Table 4).

**Table 3:** Serum immunologic profile and clinical symptoms in study population at time of diagnosis

**Variable**	**CPFE**	**IPF**
	**Number (n) – frequency (%)**	**Number (n) – frequency (%)**
Total number	40	60
ANA	17 (42.5)*	16 (26.7)
Anti-ds DNA	0	2 (3.3)
Anti-Ro (SSa)	0	0
Anti-La (SSb)	0	0
p-ANCA (MPO)	7 (17.5)*	0
c-ANCA	0	0
RF	3 (7.5)	6 (10)
Anti-CCPs	2 (5)	4 (6.6)
Anti-scl 70	1 (2.5)	2 (3.3)
Renal biopsy consistent of MPA	3 (7.5)	NA
Arthralgia	3 (7.5)	6 (10)
Morning Stiffness	2 (5)	6 (10)
Raynaud’s phenomenon	4 (10)	2 (3.3)
Dysphagia	1 (3)	2 (3.3)
Hemoptysis	2 (5)	0
Hematuria	7 (17.5)*	0

Abbreviations: *ANA*: anti-nuclear antibodies, *ANCA*: antineutrophil cytoplasm antibodies (ANCAs), *anti-CCPs*: anti cyclic citrullinated peptide antibodies, *anti-ds DNA*: anti-double strand DNA antibodies, *anti-scl 70*: anti-topoisomerase I antibodies, *IPF*: Idiopathic Pulmonary Fibrosis, *MPA*: Microscopic Polyangiitis, *MPO*: myeloperoxidase, *RF*: rheumatoid factor, *p < 0.05.

**Table 4:** Clinical, functional, survival and baseline data of patients with CPFE and ANA + ANCA + immunologic profile with and without positive renal biopsy for MPA

**Variable**	**ANA + ANCA + with positive renal biopsy for MPA**	**ANA + ANCA + without positive renal biopsy for MPA**
Patients (n)	3	4
Renal function	Renal failure	normal
Hematuria (n)	3	4
Hemoptysis (n)	2	0
Gas exchange impairment	Respiratory failure	Mild
Treatment	Pulses of methyprednisolone (1gr for 3 consecutive days) and cyclophosphamide (1gr for one day) plus oral cyclophosphamide (60 mgr/day) and oral prednisolone (60 mg/day) for 7 months	Azathioprine (2mg/kgr/day), prednisolone (20mgr/day), NAC (1800mgr/day) for 12 months
Death	1	0
FVC%pred. before Rx (∫)	60,5 ± 18,8	76,2 ± 24,5
FVC%pred. after Rx (§)	57,8 ± 17,1	70,3 ± 21,8
DL_CO_%pred. before Rx (∫)	29,5 ± 9,8	37,3 ±11,2
DL_CO_%pred. after Rx (§)	26,5 ± 10,1	35,9 ± 10,9

Abbreviations: *ANA*: anti-nuclear antibodies, *ANCA*: antineutrophil cytoplasm antibodies (ANCAs), *DL*_*CO*_: Diffusing capacity of lung for carbon monoxide, *FVC*: Forced Vital Capacity, *MPA*: Microscopic polyangiitis, *NAC*: N-acetylcysteine, *Rx*: Treatment. *p < 0.05.

∫ Pulmonary function tests 3 months before treatment initiation.

§ Pulmonary function tests after treatment initiation (7 and 12 months).

**Figure 1:**
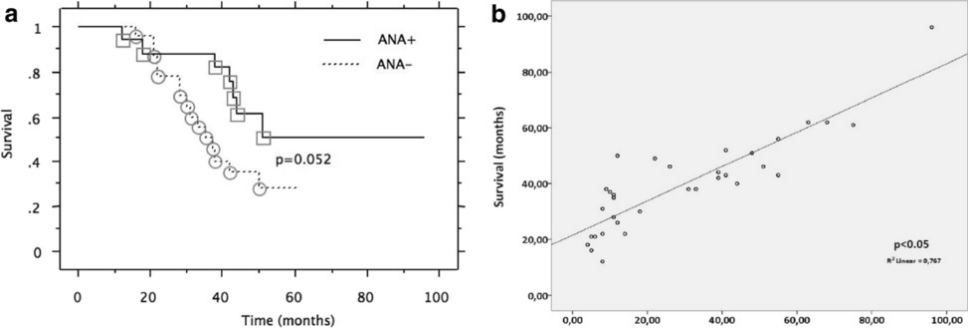
**Kaplan Meier survival and spearman’s correlation analysis in patients with CPFE with positive and negative immunologic profile. a**. Kaplan-Meier survival curve revealed a marginal statistically significant improved survival (p = 0.052) of patients with CPFE and positive ANA profile (median survival of 51 months, range 12 – 96 months) compared to those with negative immunologic profile (38 months, range 16 – 61 months) **b**. Spearman’s correlation analysis revealed an almost linear positive association between the number of CD20+ cells/mm^2^ and median survival of patients with CPFE.

There were no differences between the three groups of CPFE patients based on their immunologic profile (ANA + ANCA+, ANA + ANCA-, ANA-ANCA-) with regards to baseline functional (FEV_1_, FVC, DL_CO,_ sPAP) and radiological (HRCT pattern) parameters (Table 3). Nevertheless, Kaplan Meier analysis revealed a statistically significant improved survival of patients with CPFE and positive ANA profile compared to those with negative ANA titers (median survival 51 months-range 12–96 months vs. 38 months-range 16–61 months, p = 0.052, respectively) (Figure 1a). There were no survival differences in patients with IPF with and without positive immunologic profile (data not shown), while renal function and serologic profile returned to normal levels.

Three CPFE and 6 IPF patients with symptoms of morning stiffness and mild arthralgia exhibited marginally positive rheumatoid factor (RF) levels (>20) while one from the CPFE and two from the IPF group of patients presented with positive ENA panel as assessed by elevated circulating antibodies against topoisomerase (anti-scl70) indicative of systemic sclerosis one and two years, respectively, after the initial diagnosis of CPFE and IPF. Finally all patients exhibited negative myositis panel.

### Histopathology profile

Evaluation of the histopathology profile was performed by two independent pathologists in tissue microarray blocks derived from lung biopsy samples of 15 patients with CPFE, 28 patients with IPF and 15 controls. A pattern of definite UIP was revealed in 10 CPFE and 18 IPF cases and a probable UIP pattern in 5 CPFE and 10 IPF cases. Based on evidence arising from the investigation of the serum immunologic profile, we performed immunohistochemical analysis using CD20 antibody. The number of CD20 positive cells/mm^2^ was significantly higher in CPFE ANA + (n = 7) (49.6 ± 7.1) and IPF ANA + (n = 9) (47,1 ± 6.4) compared to CPFE ANA- (n = 8) (6.2 ± 3.6), IPF ANA- (n = 19) (5.9 ± 1.6) and control lung samples (3.2 ± 1.1) (p < 0.05) (Table 3) . There was no difference in the number of CD20+ cells between CPFE ANA + and IPF ANA + patients. Further subgroup analysis revealed that CPFE ANCA + patients with available lung biopsy samples (n = 5) also exhibited statistically significant increased CD20+ cells infiltration (51.6 ± 6.9) compared to CPFE ANCA- patients (n = 7) (7.1 ± 2.2) (p < 0.05). To further extend our previous observation that presence of positive autoimmune profile may be a positive prognosticator we performed Spearman’s correlation and clearly demonstrated an almost linear positive association between the number of CD20+ cells/mm^2^ and median survival in patients with CPFE (Figure 1b). There were no similar correlations in patients with IPF (data not shown).

**Figure 2:**
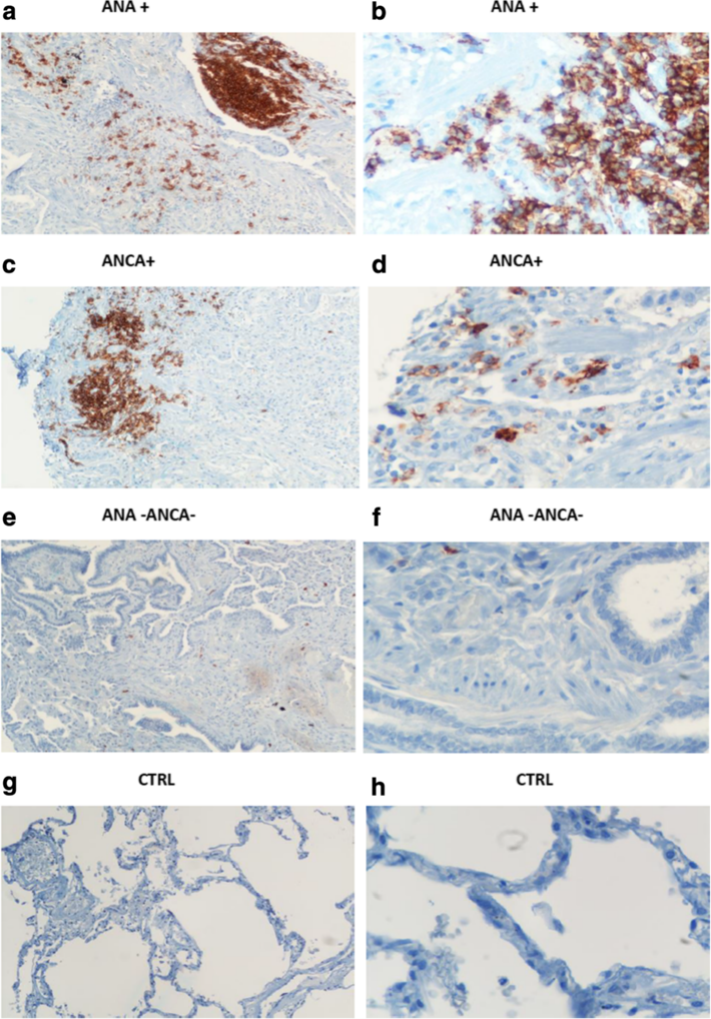
**Presence of CD20+ B cells within the fibrotic interstitium in patients with CPFE.** Representative immunohistochemistry with a CD20 antibody on lung paraffin sections from patients with CPFE with a UIP histopathologic pattern and controls. As illustrated clusters of CD20+ B cells forming lymphoid follicles were found within the fibrotic interstitium in areas adjacent to fibroblastic foci in CPFE patients with ANA + profile (**a**, **b**) as well as CPFE patients with ANCA + profile (**c**, **c**), whereas CD20+ cells were almost absent in biopsy samples from patients with CPFE and negative ANCA and ANA profile (**e**, **f**), as well as in control lung samples (**g**, **h**). Original magnification : ×200 (**a**, **c**, **e** and **g**), × 400 (**b**, **d**, **f** and **h**).

Finally and most intriguingly, as depicted in Figure 2, CD20+ B cells forming lymphoid follicles were found within the fibrotic interstitium in areas adjacent to fibroblastic foci in both CPFE ANA + (A, B) and CPFE ANCA + (C, D) lung samples whereas CD20+ cells were almost absent in CPFE ANA-ANCA- (E, F) as well as in control lung samples (G, H), supporting the presence of auto-antibody producing cells. There was no difference in CD20 + cells/mm^2^ between CPFE ANA + (49.6 ± 7.1) and CPFE ANCA + (51.6 ± 6.9) patients.

## Discussion

This is the first study in the literature investigating on a retrospective basis the immunologic profile of a relatively large cohort of patients with CPFE and comparing it to that observed in patients with IPF without emphysema, using a complete panel of clinical, serum and histopathologic markers. Our main findings are the following: 1) An increased number of CPFE patients with positive ANA profile (17/40, 42.5%) compared to patients with IPF without emphysema (16/60, 26.6%) (p < 0.05). 2) An increased number of CPFE patients with elevated serum p-ANCA titers (7/40, 17.5%) compared to none with IPF without emphysema (p < 0.05). The latter finding was accompanied by an increased prevalence of MPA (3 out of 7 patients with positive p-ANCA profile) based on renal biopsy showing pauci-immune necrotizing glomerulonephritis coupled with diffuse alveolar hemorrhage while in the remaining 4 patients immunological (positive p-ANCA titers) clinical (mild dyspnea on exertion) and serological (microscopic hematuria with normal renal function and 24-hour urine protein concentrations) findings were suggestive of an occult autoimmune disorder compatible with systemic vasculitis namely MPA. 3) A statistically significant improved survival of patients with CPFE and positive autoimmune profile compared to those with negative one suggesting that presence of autoimmunity may be associated with favorable prognosis. 4) Massive infiltration of clusters of CD20+ cells forming lymphoid follicles immediately adjacent to areas of fibroblastic foci in lung biopsy samples from CPFE patients with positive serum immunologic profile (ANCA + and/or ANA+) compared to patients with negative profile, suggesting the presence of antibody producing B cells within the injured lung. 5) A linear positive correlation of CD20+ cells with median survival in patients with CPFE indicating this histopathologic marker as a potentially reliable disease prognosticator.

CPFE represents a distinct underecognised entity seminally defined by Cottin et al. [1-5] as a syndrome characterized by frequent paraseptal emphysema with upper zone predominance imaging changes consistent with lower lobe fibrosis (UIP pattern in most cases), relatively preserved lung volumes contrasting with disproportionally impaired gas exchange, severe exercise hypoxemia and poor prognosis especially when pulmonary hypertension is present. Despite a characteristic functional and radiological profile it is still debatable whether CPFE represents a distinct syndrome or a coincidence of pulmonary fibrosis with emphysema since no common pathogenetic mechanisms have been drawn to encompass both disease entities.

Seminal observations regarding common pathogenetic pathways between emphysema and lung fibrosis identified overexpression of key molecules including tumor necrosis factor alpha (TNFa), platelet derived growth factor (PDGF) and metalloproteinases (MMPs) 2, 7, 9 as critical events leading to both alveolar and endothelial cell destruction as well as thickening of the alveolar septa depending on the lung zone [32,33]. Moreover, the past few years, several studies implicated autoimmune derangement and loss of immune tolerance in the pathogenesis of both COPD [13,34-36] and lung fibrosis[11,12], as indicated by a global numerical and functional impairment of T regulatory cells and increased titers of circulating anti-nuclear antibodies in patients with COPD [14,34] and pulmonary fibrosis [1,12,37]. Further extending the latter observations a close pathogenetic association between lung fibrosis and MPA, a type of small vessel vasculitis with renal and pulmonary involvement [38], has been recently demonstrated both in vivo and in vitro. More specifically, Tzelepis et al. [15] reported an increased incidence of pulmonary fibrosis in patients with MPA while anti-MPO antibodies have been demonstrated to induce oxidative burst and fibroblast proliferation, thus, directly contributing to lung fibrosis [16]. Further extending these evidence, our group reported a case of a patient initially diagnosed with CPFE that suddenly developed respiratory and renal failure finally attributed to MPA with lung and renal involvement [31].

On the basis of this finding,we conducted the first study in the literature investigating autoimmunity profile in a relatively large cohort of patients with CPFE. To this end, we used a complete panel comprising of clinical, serologic and histopathologic biomarkers and we compared it to that observed in patients with IPF without emphysema. Intriguingly both disease entities presented with a distinct immunologic profile with patients with CPFE characterized by an increased prevalence of positive ANA and p-ANCA serum titers. Additionally, elevated circulating anti-MPO concentrations were accompanied by a relevant clinical phenotype compatible with either a typical diagnosis of MPA with pulmonary involvement as assessed by positive renal biopsy and diffuse alveolar alveolar hemorrhage leading to respiratory failure or a more occult clinical phenotype encompassing asymptomatic microscopic hematuria with normal renal function and mild hypoxemia. Additional subgroup analysis of CPFE patients with positive immunologic profile revealed that there was no difference in terms of respiratory functional status as well as radiological and histopathological pattern between subjects with ANA+, ANCA + or ANA-ANCA- profile (Table 3). However, Kaplan-Meier survival analysis revealed that the presence of autoimmunity may be linked with a more favorable prognosis since patients with CPFE and positive ANA profile exhibited a statistically significant improved survival compared to those with negative profile (median survival of 51 vs. 38 months, p = 0.052, respectively). The latter observation is line with previous reports showing that signs and serological evidence of autoimmune disease may identify a group of patients with NSIP exhibiting improved survival [39,40].

Investigating beyond this observation, representative lung biopsy specimens from CPFE patients, with and without positive serum immunologic profile, were immunostained with CD20, a marker of B cell presence. Intriguingly, CD20+ B cells forming lymphoid follicles were found within the fibrotic interstitium in areas adjacent to fibroblastic foci in CPFE patients with positive serum ANA and p-ANCA profile whereas CD20+ cells were almost absent in biopsy samples from patients with CPFE and negative ANA profile as well as in control lung samples. In addition, an almost linear positive correlation between the number of CD20+ cells/mm^2^ of lung tissue and median survival in patients with CPFE was also demonstrated (Figure 1b) evidence that further supports the premise that serological and histopathologocal autoimmune markers may be used as reliable disease prognosticators. Nonetheless, it is worth noting that this is a retrospective study enrolling patients under different treatment arms and therefore a close association between positive autoimmune markers and disease survival cannot be safely drawn. Further, prospective larger and well designed studies using highly defined patients are sorely needed to prove such hypothesis.

The presence of B cells, immediately adjacent to fibroblastic foci in CPFE patients with positive immunologic profile, gives credence to the view that in certain cases development of both fibrosis and emphysema may be associated with massive B cell infiltration that migrate into the lung and are directed at infectious or apoptotic material derived from repetitive injurious stimuli including smoking and/or viruses. B cell follicular structures then arise and give birth to germinal centers enabling B cell isotype switching and antibody production. While on-going smoking exposure, chronic lung inflammation and lung injury proceed circulating neutrophils via endogenous signals such as, IL-1 and other inflammatory cytokines become primed and express intracellular antigens, such as MPO on their surfaces [41,42]. This process may last for prolonged periods of time even after smoking cessation and may ultimately lead to the unintended recognition of self-antigens by somatically hypermutated antibodies including ANA and/or p-ANCA that were originally targeted to non self-antigens. Circulating MPO-ANCA antibodies then attach to the primed neutrophil membranes promoting their destruction and the release of reactive oxygen species located within the neutrophilic cytoplasmic domain. The latter results in an uncontrolled oxidative burst within the pulmonary interstitium that could promote alveolar epithelial and endothelial cell apoptosis and trigger fibroblast proliferation, as it has been clearly demonstrated with MPO-induced advanced oxidation protein products from patients with MPA [16]. Additionally direct cytotoxicity of MPO-ANCA antibodies in endothelial and epithelial cells as well as fibrogenicity in resident lung fibroblasts has also been proposed [16,41,42]. Moreover, the aforementioned secondary protease-antiprotease imbalance derived from neutrophil degranulation may explain focal alveolar epithelial and endothelial cell destruction leading to emphysema [36].

Finally, the presence of a distinct histopathologic feature (CD20+ lymphoid follicles) that closely correlates with serum immunologic profile and clearly differentiates patients with CPFE into different groups (Table 3), with distinct prognostic patterns (Figure 1a), is of major importance since it may identify a specific subgroup of CPFE patients with an asymptomatic ongoing autoimmune process that may benefit from targeted immunomodulatory or immunosuppressive therapeutic approaches. To this end, CPFE patients exhibiting elevated titers of p-ANCA without positive renal biopsy (n = 4) but accompanied by an insidious clinical phenotype resembling to systemic vasculitis, with mild hypoxemia and asymptomatic microscopic hematuria and normal renal function and 24-hour urine protein levels were switched from pirfenidone treatment to low doses of corticosteroids, azathioprine and NAC. Due to the presence of relatively mild renal and pulmonary involvement, we decided to apply a more conservative therapeutic regimen and closely monitor renal and lung function rather than implementing an aggressive treatment approach with pulses of cyclophosphamide and methylprednisolone or anti-CD20 biological agents. Given their significant side-effects the application of the aforementioned therapeutic agents could be limited for more aggressive types of disease or otherwise in the context of large randomized control trials in selective groups of CPFE patients. Preliminary follow-up data seems promising since all patients (n = 4) exhibited satisfactory treatment response as assessed by both clinical and functional stabilization (Table 4) as well as improved survival.

Despite relative enthusiasm arising from the above data our study exhibited a number of limitations that should be addressed cautiously before rigid conclusions can be drawn. Firstly and most importantly, this is a retrospective study presenting with its original caveats and therefore it is hard to delineate a temporal relationship between presence of auto-antibodies and specific clinical, radiological and histopathological phenotypes. Secondly, based on the study design it is impossible to prove a causal-effect relationship between circulating auto-antibodies and B cells infiltration with alveolar epithelial and endothelial cell destruction leading to both lung fibrosis and emphysema. Our results are more suggestive of the incidence of autoimmune markers in patients with CPFE rather than supportive of a specific pathogenetic linkage. Whether CD20+ lymphoid follicular areas within the fibrotic lung represent a causal event or a compensatory protective response against fibrogenesis constitutes the subject of ongoing in vitro and experimental studies. Thirdly and most importantly patients with CPFE exhibiting only ANA positivity could simply reflect false-positivity – and not convey any autoimmune etiology. Finally, it is important to underline the fact that there is a significant background prevalence of ANA positivity in similar groups of otherwise healthy individuals and therefore the presence or absence of autoimmunity should not be based solely on non-specific serologic markers [43]. In line with the aforementioned observations ANA positivity may simply reflect loss of immune tolerance and not a flare of autoimmunity. A complete panel of clinical and serological markers should be administered in order to rigidly define the diagnosis of autoimmune disease.

## Conclusions

Collectively this is the first study in the literature reporting an increased incidence of non-specific as well as highly specific autoantibodies for systemic vasculitis with renal and pulmonary involvement namely MPA in patients with CPFE compared to patients with IPF without emphysema. Most intriguingly more than half of these patients presented with normal renal function supporting the notion that an occult autoimmune disorder with multi-organ manifestations may reside insidiously under the diagnostic “cover” of interstitial lung fibrosis and emphysema. Autoimmunity involvement was further verified by intriguing histopathologic data revealing lymphocytic infiltrates of CD20+ B cells adjacent to areas of fibroblastic foci within the fibrotic lung. In addition presence of autoimmunity was strongly associated with favorable prognosis identifying group of patients with distinct survival patterns. CPFE occurring in the context of MPA may provide a common pathogenetic mechanism for both emphysema and pulmonary fibrosis. Detailed investigation of serum immunologic profile and histopathology assessment using a panel of markers of autoimmune activity, may help us to timely identify a group of patients with unclassified autoimmune disorders which may benefit from targeted immunosuppressive therapeutic interventions including anti-CD20 monoclonal antibody. In view of the current disappointing survival data arising from large prospective placebo-controlled clinical trials, the latter evidence is of vital importance. Further well designed prospective controlled studies are sorely needed to prove this premise.

## Competing interests

The authors declare that they have no competing interests.

## Authors’ contribution

AT, MF, DB were involved in study conception and design, data acquisition and interpretation. GZ, PN, PB, PS, AA, GM were involved in patients’ recruitment and data acquisition. AO performed HRCT data assessment. AK performed histopathologic profile evaluation. MB was involved in serologic data acquisition. DM was involved in lung biopsy samples acquisition. All authors read and approved the final version of the article.

## Pre-publication history

The pre-publication history for this paper can be accessed here:

http://www.biomedcentral.com/1471-2466/13/31/prepub
